# Preparing for national school-based deworming in Kenya: the validation and large-scale distribution of school questionnaires with urinary schistosomiasis

**DOI:** 10.1111/j.1365-3156.2011.02829.x

**Published:** 2011-07-18

**Authors:** Jimmy Kihara, Charles Mwandawiro, Beth Waweru, Caroline W Gitonga, Simon Brooker

**Affiliations:** 1 Eastern and Southern Africa Centre of International Parasite Control, Kenya Medical Research InstituteNairobi, Kenya; 2 Division of Vector Borne Diseases, Ministry of HealthNairobi, Kenya; 3 Deworm the WorldNairobi, Kenya; 4 KEMRI-Wellcome Trust Collaborative ProgrammeNairobi, Kenya; 5 Department of Infectious and Tropical Diseases, London School of Hygiene and Tropical MedicineLondon, UK

**Keywords:** *Schistosoma haematobium*, schistosomiasis, school-based questionnaires, diagnosis, blood in urine, Kenya

## Abstract

**Objective:**

School questionnaires of self-reported schistosomiasis provide a rapid and simple approach for identifying schools at high risk of *Schistosoma haematobium* and requiring mass treatment. This study investigates the reliability of school questionnaires to identify such schools and infected children within the context of a national school-based deworming programme in Kenya.

**Methods:**

Between November 2008 and March 2009, 6182 children from 61 schools in Coast Province, Kenya were asked by an interviewer whether they had blood in urine or urinary schistosomiasis (kichocho), and their results were compared with results from microscopic examination of urine samples. Subsequently, in 2009, a school-based questionnaire survey for self-reported schistosomiasis was distributed by the Ministry of Education to all schools in Coast Province, and its results were compared against results from the parasitological survey. The questionnaire survey results were linked to a schools database and mapped.

**Results:**

Prevalence of self-reported blood in urine was lower among girls than boys among all ages. The use of a 30% threshold of reported blood in urine was both highly sensitive (91.7%) and specific (100%) in identifying high (>50%) prevalence schools in Coast Province. Questionnaires were however less reliable in diagnosing *S. haematobium* infection in individuals, particularly among young girls. Comparable levels of reliability were observed when the questionnaire was distributed through the existing education systems and administered by class teachers.

**Conclusions:**

The results confirm that blood in urine questionnaires can be reliably used to target mass treatment with praziquantel at national scales. The mapped results of the Ministry of Education survey serve to describe the spatial variation of urinary schistosomiasis and identify schools requiring mass treatment.

## Introduction

In recent years, an increasing number of African countries have launched national school-based deworming programmes (Garba *et al.* 2006; Kabatereine *et al.* 2006; Tchuente-Tchuenté & N’Goran 2009). In Kenya, in 2008, the education and health sectors jointly established a national school-based deworming programme. In this, mass treatment with mebendazole is provided to all school children in districts identified as having a high prevalence of soil-transmitted helminth (STH) infection (Chunge *et al.* 1985; Brooker *et al.* 2009b). For schistosomiasis, the well-established focal distribution of infection necessitated that treatment with praziquantel is targeted only to schools with a high prevalence of infection. In western Kenya, schistosomiasis is predominantly caused by *Schistosoma mansoni* (Brooker *et al.* 2009b), and previous studies indicate that there is a direct relationship between the prevalence of *S. mansoni* and distance to Lake Victoria (Brooker *et al.* 2001; Handzel *et al.* 2003), such that schools within 5 km from the lakeshore can confidently be provided with mass treatment (Brooker *et al.* 2001). On the coast of Kenya, schistosomiasis is exclusively caused by *S. haematobium*; whilst in other endemic regions of the country, both *S. mansoni* and *S. haematobium* occur. To identify schools with a high prevalence of *S. haematobium*, school-based questionnaires administered by teachers reliably identified high prevalence schools in a number of settings (Lengeler *et al.* 2002; Brooker *et al.* 2009a). Questionnaire surveys are now therefore recommended as a first step in implementing national schistosomiasis control (WHO 1995) and have to date been implemented at national or sub-national scales in Cote d’Ivoire (N’Guessan *et al.* 2007) and Tanzania (Clements *et al.* 2008a, b). In implementing a questionnaire survey, it is essential that parasitological validation should precede any large-scale use of questionnaires (WHO 1995, 2006). Here, we present the first-ever validation of school-based schistosomiasis questionnaires in Kenya and thereby contribute to the evidence base for the implementation of national deworming in the country.

## Methods

This evaluation included two components. First, as part of a larger parasitological survey (Simon Brooker, Carol Gitonga, Jimmy Kihara and Charles Mwandawiro, unpublished), a sample of 6182 children in 61 schools in Coast Province were asked by a research team whether they had blood in urine, and their answers compared with individual results of microscopy for *S. haematobium* eggs. This evaluation, conducted between September 2008 and March 2009, sought to evaluate whether blood in urine was a reliable indicator of infection in identifying both high prevalence (>50%) schools and infected individuals. Here, the same 20 children were questioned about blood in urine and asked to provide a urine sample, which was parasitologically examined for eggs of *S. haematobium*, and the sensitivity of reported blood urine was evaluated at the school and the individual level. In the second phase, a blood in urine questionnaire was distributed by the Ministry of Education to all schools in Coast Province between May 2009 and November 2009 and administered by class teachers. Results from this survey were also compared with the results from the parasitological survey conducted September 2008 to May 2009.

### The Kenyan national control programme

A number of pilot helminth control programmes have previously been undertaken in Kenya (King *et al.* 1990; Magnussen *et al.* 2001; Miguel & Kremer 2004), but without an establishment of a national framework for school-based parasite control. To help lead the way for a national programme, in 2001, the Eastern and Southern Africa Centre of International Parasite Control (ESACIPAC) was established at Kenya Medical Research Institute (KEMRI), with support from the Hashimoto initiative, Japan. A pilot school health programme was initiated in 86 schools in Mwea District in central Kenya (Kihara *et al.* 2007) and helped identify key components of a national school health programme. In 2005, the Kenya education sector constituency initiated the Kenya Education Sector Support Programme (KESSP), whose overarching goal is of enhancing access, equity and quality at all levels of education and training. To realize this goal, the programme established 23 investment programmes, one of which is the School Health, Nutrition and Feeding Investment Programme. The aim of this programme is to provide school-based health, hygiene and nutrition skills and services to all school children. In 2008, implementation funds for school-based deworming were provided by the Ministry of Education, whilst technical and operational support and drugs were provided by Deworm the World. In 2009, 3.4 million school children received mebendazole treatment for STH infection, and preparations were made for the targeted distribution of praziquantel in schools where schistosomiasis is prevalent.

### Parasitological survey

**Figure 1:**
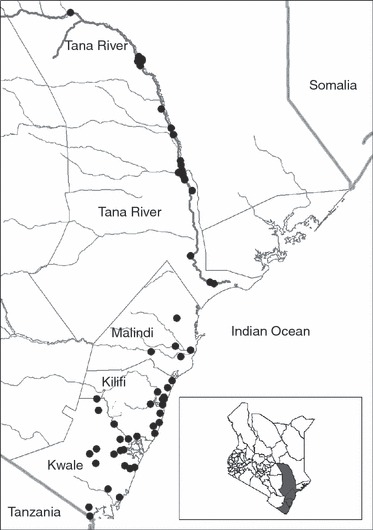
Map of the 61 schools in Coast Province, Kenya included in the parasitological survey and the main rivers, with Tana River highlighted. Insert shows map of Kenya and survey districts shaded in grey.

The parasitological survey was conducted in 61 schools in eight of the 11 districts of Coast Province (Kilifi, Kinango, Kwale, Malindi, Kaloleni, Msambweni, Tana Delta and Tana River districts) (Figure 1). These nine districts were formally a part of four larger districts (Kwale, Kilifi, Malindi and Tana River) in 1999 before district boundaries were revised throughout the country. A random selection of schools was based on the 1999 boundaries and population proportionate sampling based on the number of schools in each district. In each school, 10 boys and 10 girls (plus a reserve boy and reserve girl) were selected from each of classes 2–6 using random table numbers. A fresh urine specimen was collected from each child between 10.00 and 14.00 h to coincide with the peak production of eggs by the blood fluke *S. haematobium*. Up to 10 ml of urine was filtered through a 13-mm-diameter polycarbonate membrane with a 12-μm pore size, and the number of eggs of *S. haematobium* eggs were counted and expressed as eggs/10 ml of urine. A questionnaire of reported health problems was developed based on the questionnaires developed by the Red Urine Study Group (1995) and the Partnership for Child Development (1999). Each child was individually asked by a research team member whether he or she had experienced a number of health problems in the last 2 weeks, including headache, malaria, stomach ache, as well as whether he or she had passed blood in urine or had experienced kichocho (schistosomiasis).

Ethical clearance for the parasitological survey was obtained from the Kenya Medical Research Institute-National Ethics Review Committee in Kenya. Meetings were held with education officials, the teachers and parents to explain the purpose of the study and to obtain approval for the study. Parents who did not want their children to participate in the study were free to refuse participation. Assent was also obtained from the children before samples were collected. This passive, opt-out method of parental permission is considered to be an ethical and practical way of informing participants in low-risk studies (Ellickson & Hawes 1989), especially research used to directly guide government public health intervention.

### Analysis of diagnostic performance

Reported schistosomiasis (kichocho) was not readily understood by children and therefore not investigated here. Associations at the school level were investigated by plotting the prevalence of reported blood in urine against the prevalence of *S. haematobium* infection. Previous analyses (Red Urine Study Group 1995; Guyatt *et al.* 1999; Ansell *et al.* 2001) suggest that a prevalence of reported blood in urine ≥30% is equivalent to the prevalence of *S. haematobium*≥50%. On this basis, sensitivity was calculated as the percentage of schools with the prevalence of *S. haematobium*≥50% and reported blood in urine ≥30%, and specificity was calculated as percentage of schools with the prevalence of *S. haematobium* <50% and reported blood in urine <30%. The negative predictive value (NPV) and positive predictive value (PPV) of the 30% threshold prevalence of reported blood in urine in identifying low and high prevalence schools for *S. haematobium* were calculated as follows: NPV = percentage of schools with a reported blood in urine <30%, which have a prevalence of *S. haematobium* <50%; PPV = percentage of schools with a reported blood in urine ≥30%, which have a prevalence of *S. haematobium*≥50%, and 95% exact binomial confidence intervals (CIs) were calculated.

Children in the parasitological survey were classified into four categories: true positives (self-reported blood in urine and eggs seen in urine); true negatives (blood in urine not reported and no eggs seen in urine); false positives (self-reported blood in urine but no eggs seen in urine); and false negatives (blood in urine not reported but eggs seen in urine). The performance of reported blood in urine in identifying children infected with *S. haematobium* was assessed according to sex and age group (<10, 10–12 and 13+ years) in terms of sensitivity (the percentage of infected children who reported blood in urine) and specificity (the percentage of uninfected children who did not report blood in urine). Positive and negative predictive values were also calculated. All values and their 95% exact binomial CIs were calculated using the *diagt* command in Stata version 10.0 (StataCorp, College Station, TX, USA), and non-overlapping CIs were indicative of a statistical difference between age and sex groupings. Significant differences between prorportions were tested using logistic regression and correlations assessed with a Spearman rank test.

### Government questionnaire survey

Between May and November 2009, questionnaires were delivered to the District Education Officers (DEO) in all districts of Coast Province. Subsequently, questionnaires were distributed to the head teachers of each school along with instructions on how class teachers should implement the questionnaire. The questionnaire was the same one used in the parasitological survey and included 15 health-related problems. On the day of the survey, each child present was asked by the class teacher whether he/she had experienced any of the health problems in the last 2 weeks. Class totals were collated by the head teachers, and the questionnaires were to be returned to the DEO at the end of the month when the head teachers went to collect salaries. The questionnaires for each district were then passed on to the national programme office in Nairobi. School results were linked to a national school database developed for the Ministry of Education (Ministry of Education 2008) and mapped using ArcGIS 9.3 (ESRI, Redlands, CA, USA).

Of the 61 schools included in the parasitological survey, only 45 (74%) returned the questionnaire. For these schools, associations at the school level were investigated simply by plotting the prevalence of reported blood in urine against the prevalence of *S. haematobium* infection. The NPV and PPV of a reported threshold prevalence of blood in urine in identifying low and high prevalence schools for *S. haematobium* were calculated as follows: NPV = percentage of schools with a reported blood in urine <30%, which have a prevalence of *S. haematobium* <50%; PPV = percentage of schools with a reported blood in urine ≥30%, which have a prevalence of *S. haematobium*≥50%.

## Results

**Figure 2:**
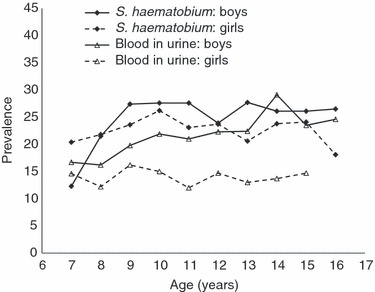
Prevalence of *Schistosoma haematobium* and reported blood in urine by age group and sex among 6182 school children in 61 schools in Coast Province, Kenya, 2008–2009.

Sixty-one schools, which included 6182 children aged 5–20 years, were selected for the parasitological study. The number of children in each school ranged from 79 to 113 children (median 105), with a similar number of boys (3077) and girls (3105) sampled. The overall prevalence of infection with *S. haematobium* was 24.5% [95% CIs: 23.4–25.6%], with prevalence ranging from 0 to 91.3% among schools. Prevalence of infection initially increased with age, then flattened out (Figure 2), and boys were significantly more infected than girls (26.0%*vs.* 23.0%, *P* = 0.006). Overall, 18.2% (95% CIs: 17.2–19.2%) of children reported blood in urine, with more boys reporting than girls (22.5%*vs.* 13.9%, *P* < 0.001). The prevalence of reported blood in urine increased with age among boys, but was constant with age among girls (Figure 2).

**Figure 3:**
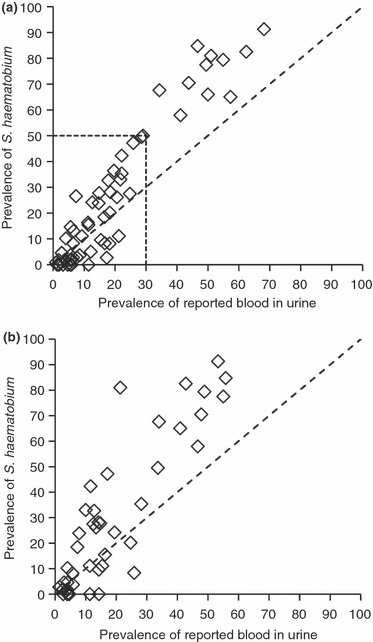
(a) Relationship between the prevalence of infection with *Schistosoma haematobium*, diagnosed by microscopy of urine, in children among 6182 children in 61 primary schools along the coast of Kenya and along the Tana River, 2008–2009, and the prevalence of reported blood in urine among the same children determined during an interview with a research team member. (b) Relationship between the prevalence of infection with *S. haematobium*, diagnosed by microscopy of urine, in the 27 schools who returned the Ministry of Education questionnaire, and the prevalence of reported blood in urine among all children in the same schools. The dotted line indicates equivalence of prevalences. Box indicates those schools with prevalence of reported blood in urine <30% and prevalence of *S. haematobium* <50%; outside the box are schools with prevalence of reported blood in urine ≥ 30% and prevalence of *S. haematobium*≥ 50%.

Figure 3a shows the relationship in the 61 schools between the prevalence of urinary schistosomiasis diagnosed by urine microscopy and the prevalence of reported blood in urine. There was a significant correlation between these prevalences (*r* = 0.94, *P* < 0.001), although the figure shows that reported prevalence of blood in urine underestimated the prevalence of infection by an approximately consistent amount, over the range of prevalences observed. This relationship confirms that reported blood in urine is a reliable indicator of moderate and high prevalence schools. For instance, using a threshold of 30% reported blood in urine to identify high (>50%) schools yields a sensitivity of 91.7% (95% CIs: 61.5–99.7%) and a specificity of 100% (95% CIs: 92.7–100%) (Table 1). The ability of reported blood in urine to identify infected individuals is also reported. Table 1 indicates that questioning children about blood in urine has very poor sensitivity (48.9%) but high specificity (91.8%). Sensitivity was significantly better among boys (57.8%) than among girls (39.0%), and among boys, sensitivity marginally increased with increasing age, whilst among girls, sensitivity was highest among girls aged 10–12 years old.

**Table 1:** The sensitivity, specificity, positive predictive value (PPV) and negative predictive value (NPV) of reported blood in urine in (a) identifying school with prevalence >50% and (b) in diagnosing children infected with *Schistosoma haematobium* on the Kenyan coast, 2008–2009. Exact 95% binomial confidence intervals in parenthesis

	Overall
(a) Schools	
Sensitivity	91.7 (61.5–99.7)
Specificity	100 (92.7–100)
PPV	100 (71.5–100)
NPV	98.0 (89.3–99.9)

(b) Children				
	All ages	Age <10 years	Age 10–12 years	Age 13+ years
Boys				
Sensitivity	57.8 (54.2–61.2)	52.9 (42.8–62.9)	57.5 (52.1–62.7)	59.4 (54.1–64.6)
Specificity	90.0 (88.7–91.2)	92.3 (88.9–94.9)	90.9 (89.0–92.7)	88.2 (86.0–90.2)
PPV	67.0 (63.3–70.5)	67.5 (56.1–77.6)	69.2 (63.5–74.5)	64.8 (59.3–87.7)
NPV	85.8 (84.4–87.2)	86.7 (82.7–90.0)	85.8 (83.5–87.8)	85.6 (83.2–87.7)
Girls				
Sensitivity	39.0 (35.4–42.7)	35.2 (26.8–44.4)	40.5 (35.2–45.9)	38.8 (32.7–45.1)
Specificity	93.6 (92.6–94.6)	91.5 (88.4–94.0)	94.5 (93.0–95.8)	93.8 (91.8–95.1)
PPV	64.7 (59.9–69.2)	54.4 (42.8–65.7)	70.4 (63.5–76.7)	62.6 (54.5–70.2)
NPV	83.7 (82.3–85.1)	83.0 (79.3–86.3)	83.2 (80.9–85.2)	84.7 (82.3–86.9)

**Figure 4:**
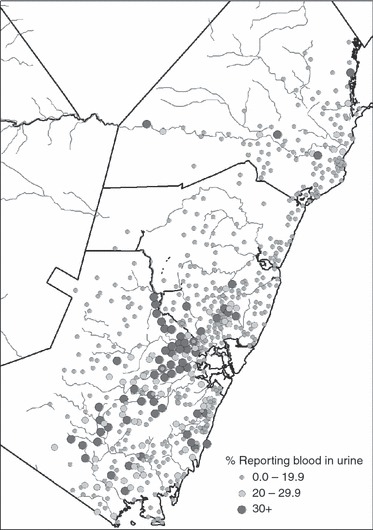
The spatial distribution of reported blood in urine in 630 schools on the coast of Kenya based a questionnaire distributed by the national school-based deworming programme, 2009.

As a part of the national control programmes, questionnaires were targeted for distribution to 1772 schools. Of the 61 schools with parasitological data, 45 (74%) schools returned the completed questionnaires. Figure 3b shows the relationship in these 45 schools between the prevalence of *S. haematobium* diagnosed by urine microscopy and the percentage of all children reporting blood in urine among those who responded as a part of the Ministry of Education questionnaire. There was a significant correlation between these prevalences (*r* = 0.91, *P* < 0.001). Using a threshold of 30% reported blood in urine to identify high (>50%) schools, yielded a sensitivity of 90.0% (95% CIs: 54.4–99.7%) and a specificity of 97.1% (95% CIs: 85.1–100%). Figure 4 presents the spatial distribution of reported blood in urine based on the Ministry of Education questionnaire survey and highlights the marked focality of infection, with the highest prevalence occurring in Kwale District and in Mariakani of Kilifi District.

## Discussion

Scaling-up of schistosomiasis control throughout sub-Saharan Africa serves to emphasize the role of operational research to ensure that programmes are implemented appropriately and cost-effectively. Both the focal distribution of schistosomiasis and the relatively high cost of praziquantel require that treatment is targeted to schools and communities at great risk of infection and disease in endemic areas. The data presented here show that within the context of a national deworming programme in Kenya, school questionnaires can reliably identify such high prevalence schools. The observed high sensitivity and specificity are consistent with research studies conducted elsewhere (Lengeler *et al.* 2002; Nduka & Nwosu 2008).

The lower prevalence of reported blood in urine and the poor performance of reported blood in urine in identifying infected individuals, especially among older girls, corroborate with previous studies (Red Urine Study Group 1995; Lengeler *et al.* 2002; Clements *et al.* 2008a). For instance, a survey among Tanzanian school children found that 28.2% of girls reported blood in urine compared with 48.6% of boys and that reported blood in urine had a sensitivity of 51.5% and a specificity of 79.2%, but sensitivity was significantly lower in girls than in boys (39.6%*vs.* 61.9%) (Guyatt *et al.* 1999). Also in Tanzania, a 2004 national questionnaire survey was administered to over 2.5 million children in 12 399 schools across the country (Clements *et al.* 2008b). Analysis of differences of reported blood in urine by age and sex found that whilst the prevalence of *S. haematobium* increased with age among girls, the prevalence of reported blood in urine either flattened out or decreased, indicating the latter underestimated prevalence in older girls (Clements *et al.* 2008a). It has been suggested that the onset of menses influences girls’ willingness to report blood in urine.

Four main limitations are identified in the present study. First, the use of a single urine filtration may have underestimated the prevalence of *S. haematobium* infection; however, the magnitude of the underestimation is unlikely to have lead to a gross misclassification of schools. Second, there is slight temporal disconnect between the time when the parasitological data were collected (September 2008 to March 2009) and when the questionnaire was distributed by the Ministry of Education (May to November 2009), which may potentially reduce the validity of the validation of the government questionnaire survey. However, no mass treatment was undertaken between the parasitological survey and the government questionnaire survey, and although there may have been seasonal fluctuations in the two free-living aquatic stages, the miracidia and the cercaria, and of the infected snail hosts, it is unlikely that there would be significant fluctuations in the prevalence of human infection. This is because the life span of adult worms is substantially longer (3–6 years) than those of either infected snail hosts (weeks) or free-living stages (hours). A third limitation, and one related to the second limitation, is that individuals found to be infected in the parasitological surveys were treated and so may have contributed to an underestimation of reported blood in urine as a part of the government survey. However, these individuals represented only a small proportion of the total school population, and mass treatment was provided to high prevalence schools only after the questionnaire survey. Fourth, only 74% of schools returned the Ministry of Education questionnaire, and this may have introduced selection bias (to an unknown degree). Future implementation of the questionnaire survey will need to identify strategies to increase the return rate.

The study is one of very few that effectively seek to target praziquantel treatment at a national level. Linking the questionnaire survey results to the national schools database enabled mapping of the spatial distribution of infection and estimation of the number of schools requiring mass treatment. This approach has now been extended to other areas of Kenya endemic for urinary schistosomiasis, including central and western Kenya. The administrative structures of the Ministry of Education turn out to be a cost-effective mechanism for distributing questionnaires. In addition, the establishment of a joint Ministry of Public Health and Sanitation and Ministry of Education school health committee, plus a steering committee of donors and stakeholder, to oversee the national deworming programme was crucial to this exercise, ensuring that health responsibilities were met and ensuring the quality of implementation through schools. These processes and features lay the foundation for future control of schistosomiasis in Kenya.
